# Construction of Ag-TiO_2_ Hierarchical Micro-/Nanostructures on a Ti Plate for Photocatalysts via Femtosecond Laser Hybrid Technology

**DOI:** 10.3390/mi14101815

**Published:** 2023-09-22

**Authors:** Qian-Kun Li, Yue Li, Yan-Jun Wang, Jin-Yong Qi, Yan Wang, Yao-Dong Liu, Xue-Qing Liu

**Affiliations:** 1 State Key Laboratory of High Power Semiconductor Lasers, School of Physics, Changchun University of Science and Technology, 7089 Wei-Xing Road, Changchun 130022, China; liqiankun@cust.edu.cn; 2 Key Laboratory of Advanced Structural Materials of Ministry of Education, Changchun University of Technology, Changchun 220103, China; liyue02204156@163.com (Y.L.); wyj13280365342@163.com (Y.-J.W.); liuyaodong@ccut.edu.cn (Y.-D.L.); 3 State Key Laboratory of Integrated Optoelectronics, College of Electronic Science and Engineering, Jilin University, Changchun 130012, China; q1j2y33@163.com

**Keywords:** femtosecond laser, hierarchical micro-/nanostructures, photocatalyst, titanium dioxide

## Abstract

Titanium dioxide photocatalysts can break down pollutants using natural light. They possess notable light stability, chemical stability, and catalytic effects, thus leading to extensive research worldwide. However, the limited light absorption range of titanium dioxide and their inefficiencies in generating and transporting photogenerated carriers hinder the enhancement of their photocatalytic performance. In this study, we employ a femtosecond laser composite processing method to create an Ag-TiO_2_ nanoplate composite catalyst. This method doubles the catalytic efficiency compared with the structure processed solely with the femtosecond laser. The resulting Ag-TiO_2_ nanoplate composite catalysts show significant promise for addressing environmental and energy challenges, including the photodegradation of organic pollutants.

## 1. Introduction

Titanium dioxide (TiO_2_) photocatalysts, owing to their cost-effectiveness, non-toxic nature, and long-term physicochemical stability, have become prominent in addressing environmental and energy challenges. These applications include photodegradation of organic pollutants [1], self-cleaning capabilities [2], air purification [3], and wastewater treatment [4]. The commendable photocatalytic activity of TiO_2_ is primarily due to the electron transitions between its conduction and valence bands, thus leading to the creation of highly reactive free radicals [5,6]. However, a significant limitation of TiO_2_ is its narrow bandgap energy (3.2 eV for anatase and 3.0 eV for rutile) [7,8], which restricts its absorption to the ultraviolet spectrum, representing approximately 4% of the earth’s solar light. This narrow absorption spectrum, along with the high propensity for photogenerated carrier [9] recombination, hinders further enhancement of its photocatalytic efficiency.

An optimal photocatalyst should possess a wide light absorption range, coupled with the efficient generation and transfer of photogenerated carriers. Currently, the two promising approaches for extending TiO_2_’s absorption capacity into the visible region are band gap modification [10,11,12] and the creation of gradient microsurfaces [13,14,15]. The band gap of TiO_2_ can be effectively broadened to the visible spectrum by doping with metals (such as Au, Ag, Fe, and Pd) [16,17,18,19] and non-metals (such as N and C) [20,21] to introduce intermediate energy levels. Concurrently, doping can infuse defects into the TiO_2_ lattice [22,23,24], thus potentially altering its crystalline state, which can suppress the recombination of photogenerated carriers. Among the precious metals, Ag stands out as a popular choice for doping owing to its affordability and accessibility, compared with the likes of Au and Pd. The integration of TiO_2_ with Ag creates a Schottky barrier [25,26,27], which functions as an electron trap, thus hindering recombination and fostering charge separation at the interface. Moreover, the surface plasmon resonance (SPR) effect generated by silver nanoparticles (NPs) significantly amplifies the absorption of visible light.

The creation of microstructures has been demonstrated to induce SPR [28,29] and enhance transfer efficiency. The femtosecond laser processing technique is renowned for its application in surface and internal modifications as well as the removal of various metallic and non-metallic materials. Its popularity can be attributed to its exceptionally short pulse duration and elevated peak energy density, combined with its capability for true three-dimensional (3D) processing [30,31,32,33,34,35]. This technology involves directing a concentrated, high-energy-density pulsed laser beam onto a localized area of the sample. This leads to material vaporization and sequential removal, thus enabling the meticulous crafting of surfaces and edges that are both complete and smooth. The resultant micro-/nanostructures amplify the specific surface area [36,37]. Furthermore, this process induces the separation of oxygen within the lattice material, thus resulting in the formation of oxygen vacancies. These oxygen vacancies play a dual role [38,39]: they act as active sites, which reduce reaction energy barriers and facilitate molecular activation [40,41], and they aid in modifying the energy band structure to decrease the bandwidth. Concurrently, they foster the transformation of excitons into carriers [42] and augment carrier separation.

In this study, we introduce a three-step methodology for the fabrication of Ag-TiO_2_ micro-/nanocomposite structures. Initially, a consistent 3D grid of micro-/nanostructures was crafted onto the Ti surface using a femtosecond laser. Subsequently, we employed hydrothermal synthesis to produce TiO_2_ micro-/nanoflakes with a richer array of morphologies. In the final step, Ag nanoparticles were incorporated chemically to establish the Ag-TiO_2_ micro-/nanocomposite structures. We assessed the photocatalytic attributes of Ag-TiO_2_ catalysts under varying laser processing parameters and concentrations. The findings revealed that using a processing power of 2.5 W, at a speed of 5 mm/s, and after 10 repetitions, coupled with a concentration of 3 mol/L, yielded the most efficient photodegradation. Remarkably, this catalyst maintained robust catalytic performance even after 90 min of usage.

## 2. Materials and Methods

### 2.1. Materials

A 99.99% pure titanium sheet, with a thickness of 0.3 mm, was procured from Baoji Yixin Titanium Industry. It was cut into 10 mm × 10 mm square slices for processing. Prior to laser treatment, the oxide layer on the titanium surface was polished sequentially with 400–2000 grit sandpaper; subsequently, it was ultrasonically cleaned with acetone, alcohol, and deionized water. The samples were dried using nitrogen and stored in a vacuum drying box. Sodium hydroxide, polyvinylpyrrolidone, and sodium borohydride were sourced from Chinese Medicine Group Reagent Co., Ltd., Shanghai, China.Methylene blue (MB) was obtained from Aladdin Chemical Reagent Co., Ltd., Shanghai, China. All chemicals, of an analytical grade, were used as received.

### 2.2. Preparation Process

**Figure 1:**
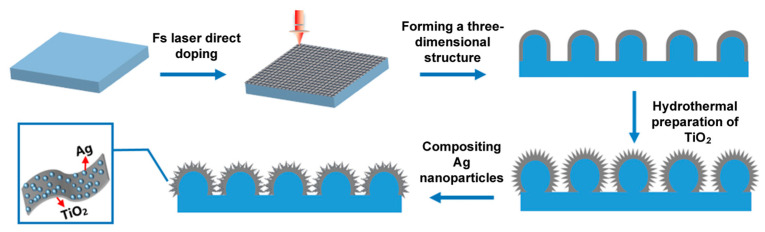
Schematic of a multistage micro-/nano-Ag-TiO_2_ structure based on Ti surface.

Firstly, femtosecond laser processing created microstructures with a uniform 3D lattice on the titanium sheets (illustrated in Figure 1). The parameters of the femtosecond laser we used are as follows: pulse width is 260 fs, pulse repetition frequency is 200 kHz, center wavelength is 1030 nm, and pulse diameter is about 5 μm. Varied structural surfaces were realized by manipulating the processing rate, power, and time. Subsequently, TiO_2_ nanowires were synthesized with a hydrothermal method at 180 °C in a NaOH solution for 30 h with different NaOH concentrations (1, 2, and 3 mol/L). The treated Ti tablets were then immersed in a 1 mol/L HCL solution for 1 h, rinsed with deionized water, and annealed at 450 °C. For Ag nanoparticle compounding, 0.2 g of polyvinylpyrrolidone was mixed in a water/ethylene glycol solution (1:1 volume ratio, 40 mL total). After stirring, 0.01 g of NaBH_4_ and 3 mmol of AgNO_3_ were added until the solution attained a brown-red hue. The femtosecond-laser-prepared TiO_2_ nanoscale was immersed in this mixture, maintained in a 40 °C oil bath for 5 h, and subsequently cleaned with deionized water, thereby resulting in the Ag/TiO_2_ composite structure.

### 2.3. Characterization

The sample’s morphology post-laser-treatment was examined using field emission scanning electron microscopy (FESEM; Zeiss SUPRA). The surface chemical composition of the sample was determined through an energy dispersive spectrometer (Genesis Apollo XL). The laser-induced grating structure was characterized using an Olympus laser confocal microscope (OLS3000). The distribution of Ag nanoparticles on the TiO_2_ nanowires was assessed via transmission electron microscopy (TEM; JEM-2100F), whereas lattice fringe analyses were conducted via a high-resolution transmission electron microscope (HRTEM). The results confirmed that the TiO_2_ type was anatase, with Ag nanoparticles integrated into the TiO_2_ nanowires, forming a micro-/nanocomposite structure. The MB solution, serving as a representative organic pollutant, was utilized at a concentration of 5 g/L. The light source for the experiment was a simulated solar system (PL-XQ500W, provided by Beijing Princes Technology Co., Ltd., Beijing, China). Photocatalytic degradation tests were performed under sunlight intensity. The efficacy of different samples was juxtaposed, with the characteristic peak changes of the MB solution at 664 nm being verified using a Shimadzu UV-2550 spectrophotometer. The photocatalytic reaction was initiated upon lamp illumination.

## 3. Results and Discussion

### 3.1. Effect of Femtosecond Laser Processing Parameters on Surface Morphology

**Figure 2:**
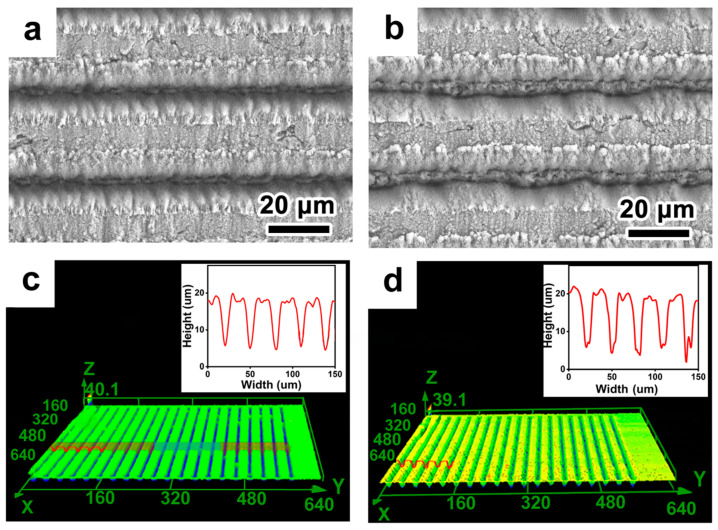
Structural morphology characterization of single and multiple laser treatments. SEM images of single processing (**a**) and 10-time processing (**b**). (**c**,**d**) are 3D morphologies of laser-fabricated structures. The insert image in (**c**,**d**) is the cross-section profile of the structures.

The femtosecond laser, when concentrated, possesses extraordinarily high energy. It can fabricate micro-/nanostructures by vaporizing the material surface at energy densities in the range of TW/cm^2^. To refine the processing parameters, we examined the influence of the number of laser scans on the grating structure, maintaining a constant laser power of 2.5 W. With a scanning spacing of 30 μm, Figure 2a depicts the surface after a single scan, whereas Figure 2b illustrates the result after 10 scans. The corresponding laser confocal 3D and profile images are represented in Figure 2c,d. As the machining duration increases, the groove depth and bottom width expand. A single scan yields a grating depth of 13.4 μm, which extends to 19.8 μm after 10 consecutive scans. Observing the unprocessed region reveals a smooth surface, with almost no discernible heat-affected zones in Figure 2a,b.

**Figure 3:**
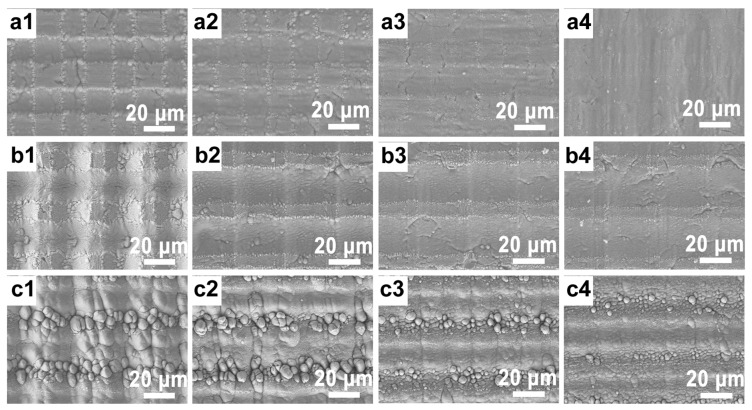
The relationship of the morphologies of laser-fabricated structures with scanning rates and laser power. (**a**) Effect of different scanning rates on surface structure at laser power of 2 W: (**a1**) 5 mm/s, (**a2**) 10 mm/s, (**a3**) 15 mm/s, and (**a4**) 20 mm/s. (**b**) Effect of different scanning rates on surface structure at laser power of 2.5 W: (**b1**) 5 mm/s, (**b2**) 10 mm/s, (**b3**) 15 mm/s, and (**b4**) 20 mm/s. (**c**) Effect of different scanning rates on surface structure at laser power of 4 W: (**c1**) 5 mm/s, (**c2**) 10 mm/s, (**c3**) 15 mm/s, and (**c4**) 20 mm/s.

Given the significant impact of scanning speed and scanning power on microstructure formation, we adjusted the laser processing power and rate to craft varied micro-/nanosurfaces on Ti wafers, as depicted in Figure 3. Maintaining a line spacing of 30 μm and processing once, the power was modulated to 2 W, 2.5 W, and 4 W. Concurrently, scanning rates were varied from 5 mm/s, 10 mm/s, and 15 mm/s to 20 mm/s. As shown in Figure 3(a1–a4), the laser power was 2 W. Observations revealed that, with a laser rate of 5 mm/s, a regular grid structure formed, with horizontal and vertical patterns mirroring the laser scanning trajectory. Unscanned portions remained untouched and smooth. The absence of a pronounced groove might be attributed to an underpowered laser, which resulted in the generation of only some sub-wavelength nanostripes on the surface. As the processing rate was incrementally increased to 10, 15, and 20 mm/s, the etching depth decreased. At 20 mm/s, the surface micro-/nanostructure became indistinguishable, likely owing to the femtosecond laser’s restricted ablation rate. Consequently, a higher rate yielded a smaller processing depth. In comparison to the 2 W power setting, the 2.5 W grid structure emerged as uniformly defined, with sporadically placed sub-wavelength nanostripes and micro-/nanoparticles evident in the spot-treated region, culminating in a cohesive prism structure, as shown in Figure 3(b1–b4). This outcome aligned with the effects observed at 2 W, when the scanning rate was similarly elevated. Notably, upon increasing the processing power to 4 W, protuberances with diameters ranging between 1 and 3 μm resulted in the spot-treated region, essentially displacing the prism structure, as shown in Figure 3(c1–c4). The non-lit spot processing area deviated from its flat demeanor. As scanning rates continually increased, the untreated surface morphed into a ridged pattern, thereby intensifying the granularity of the bulge. Of all the tested configurations, the most pronounced prismatic surface was realized at a power of 2.5 W and a rate of 5 mm/s, essentially delivering the largest specific surface area.

**Figure 4:**
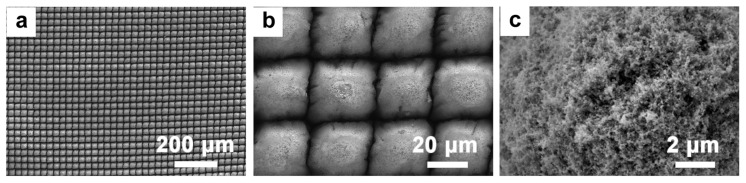
The morphologies of laser-fabricated structures with repeated processing for 10 times. (**a**–**c**) The SEM images at different magnifications.

After setting the processing power and rate to 2.5 W and 5 mm/s, respectively, the processing times were adjusted. Specifically, the procedure is repeated 10 times, as depicted in Figure 4. Figure 4a illustrates the expansive grid-like structure crafted on the Ti surface. This structure is consistent in size, with its height nearly mirroring its depth, and lacks the submicron protuberance that power fluctuations might introduce. As observed in Figure 4b,c, the peripheral region following multiple laser ablations is smooth and uniform. At the top, owing to the line spacing exceeding the spot diameter, complete etching was unattainable, thus resulting in a sleek, isolated prismatic structure interspersed with micro-/nanostripes. When contrasted with the standalone prismatic formation, the grid structure created on the Ti surface after 10 processing cycles boasted a more substantial specific surface area. Moreover, this structure could be crafted across a broader expanse, maintaining its smooth and even texture.

### 3.2. Preparation of TiO_2_ Nanosheets on Micro-/Nanosurfaces via Laser Machining

**Figure 5:**
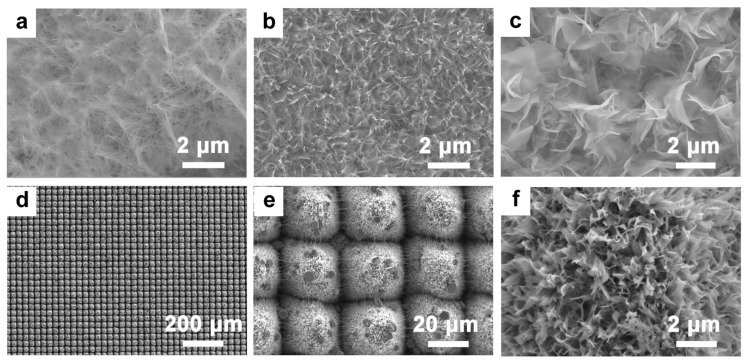
The effect of different NaOH concentrations on the morphology of nano-TiO_2_ and the morphology of the overall structure at (**a**) 1 mol/L, (**b**) 2 mol/L, and (**c**) 3 mol/L. (**d**–**f**) Morphology of TiO_2_ micro-/nanostructures.

The NaOH hydrothermal method is frequently employed to produce titanium dioxide due to its uniformity, low reaction temperature, high purity, and controllable structure. To investigate the influence of NaOH concentration on the morphology of nano-TiO_2_ on the microsurface, hydrothermal treatment was conducted on the Ti surface without prior femtosecond laser treatment. As depicted in Figure 5, the tested NaOH concentrations were 1, 2, and 3 mol/L. At a concentration of 1 mol/L, an abundance of interconnected nanowires emerged on the Ti surface. Elevating the concentration to 2 mol/L resulted in thicker nanowires with more defined boundaries. In similar magnifications, the density of the nanowires surpassed that observed in the 10 mol/L concentration. At 3 mol/L, the nanowires transitioned into large clusters of nanosheets, thus highlighting the impact of increased NaOH concentration on the nanostructure’s evolution. A closer examination of the TiO_2_ nanostructure generation revealed that during the hydrothermal treatment of Ti with NaOH, the primary processes were dissolution and recrystallization. This caused the Ti substrate to form Na_2_Ti_2_O_5_ nanocrystals, which served as the primary Ti source. As Ti atoms continuously migrated from the substrate and with ongoing nanocrystal formation, the incessant generation of Na_2_Ti_2_O_5_ nanocrystals occurred. Based on Gibbs–Curie–Wulff’s law, crystal formation tends to occur in regions with elevated surface energy, leading to vertical nanostructure growth from the substrate. With further increases in NaOH concentration, Ostwald ripening occurred. Smaller nanocrystals dissolved and reformed on larger structures. This resulted in the transformation of densely packed nanowires at lower concentrations to broader nanowires at a 3 mol/L concentration. Concluding the reaction with annealing at 450 °C ensured improved contact and elevated crystallinity between the substrate and TiO_2_ nanowires.

Different femtosecond laser processing durations influence the morphology of the titanium dioxide micro-/nanostructures, as illustrated in Figure 5d–f. Compared with the planar TiO_2_, the structure of TiO_2_ post-femtosecond-laser-processing resembled intertwined flower-like formations, particularly in the regions between the grooves. This resulted in a markedly increased specific surface area. When contrasting the structures formed after a single processing step with those formed after 10 cycles, the differences became evident. the nanoflowers resulting from the 10-cycle processing appear more distinct and less tangled, thus yielding a larger specific surface area. The increased etching depth from the 10-cycle processing might prevent excessive cross-linking, thus offering more active growth sites for the TiO_2_. A close examining of the individual nanoflower structures in Figure 5e,f reveals that the nanoflower configuration is more pronounced after 10 processing cycles. This enhanced definition results in an even greater specific surface area and increased contact with pollutants, thereby boosting the catalytic efficiency.

### 3.3. Synthesis of Silver Nanoparticles with Chemical Methods

**Figure 6:**
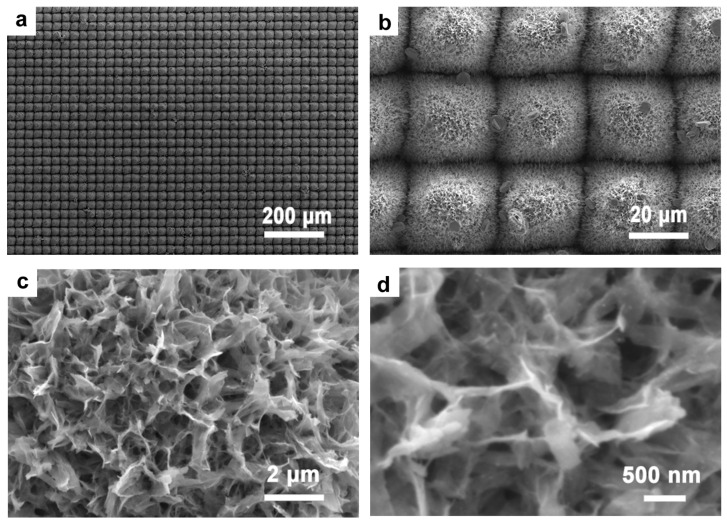
(**a**–**d**) SEM of Ag-TiO_2_ surface topography on a laser-fabricated microstructure.

**Figure 7:**
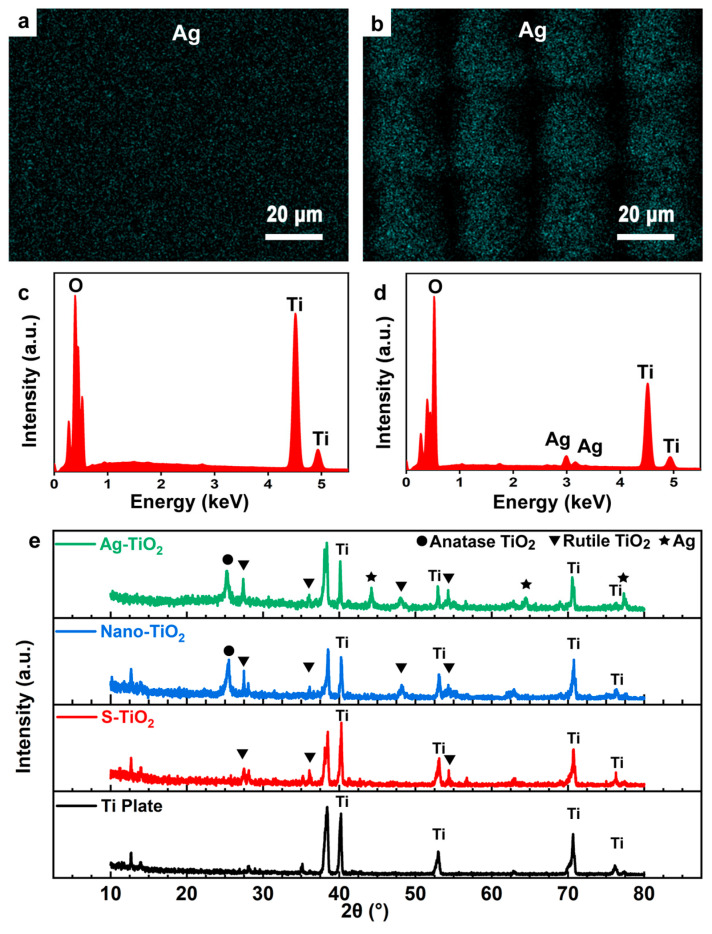
EDS mapping of untreated Ti plate (**a**) and Ag-TiO_2_ on microstructures (**b**). (**c**,**d**) The energy spectrum of the samples. (**e**) XRD spectrum of Ti plate, S-TiO_2_, nano-TiO_2_, and Ag-TiO_2_, respectively.

Ag particles were integrated with TiO_2_ nanostructures through a reduction method to produce multitiered micro-/nanostructures. Figure 6 displays the SEM images of these samples. Notably, Ag nanoparticles are scattered across the surface of the micro-/nanostructures, clustering irregularly atop the flower-like formations. The presence of cracks on the surface can be attributed to the overlapping TiO_2_ nanoflowers. Given their inherent instability, these structures are prone to cracking, possibly owing to differential thermal expansion coefficients during the Ag ion composite process. Figure 6c,d provides a high-resolution local view, essentially highlighting the TiO_2_ nanowires peppered with dense white clusters. A subsequent EDS energy spectrum analysis confirmed these clusters as Ag particles. Furthermore, the distinctive petal features become more apparent when capturing images of individual nanoflowers, as verified with EDS, as shown in Figure 7. Notably, Ag particles display a uniform distribution over the nanoflower structures. This is likely because the structures are more distinct, and during the hydrothermal process, the number of binding sites for Ag particles increases. Hence, Ag can be distributed at the base of the nanoflowers, rather than just on their surface. An EDS elemental analysis corroborates this observation, thus indicating a pronounced peak for Ag.

For clarity in descriptions, the unprocessed flat titanium sheet is labeled as Ti; the TiO_2_ structured with a micro-/nanogrid post-femtosecond-laser-processing is labeled as S-TiO_2_; the TiO_2_ surface treated with the NaOH hydrothermal method is labeled as nano-TiO_2_; and following these treatments, TiO_2_ combined with Ag nanoparticles is labeled as Ag-TiO_2_. Figure 7e shows the XRD test results of untreated titanium plates, after laser treatment, after hydrothermal reaction, and after compounding Ag nanoparticles, named Ti, S-TiO_2_, nano-TiO_2_, and Ag-TiO_2_, respectively. The untreated titanium plates showed peaks at 2θ values of 40.16 (101), 52.98 (102), 70.63 (103), and 76.19 (112), respectively. Rutile TiO_2_ appeared after laser processing and the corresponding peaks appeared at 27.4 (110), 36.1 (101), and 54.36 (111). The crystal phase of TiO_2_ after the hydrothermal reaction includes both the rutile phase with a 2θ value of 48.18 (200) and the anatase phase with a 2θ value of 24.8 (101). The two crystalline types of TiO_2_ after the hydrothermal reaction were mixed together for a better catalytic effect. Finally, regarding composite Ag nanoparticles, the peak positions of Ag appeared on the corresponding spectra with 2θ values of 44.22 (200), 64.46 (220), and 77.32 (311). According to the above XRD results, bits of rutile TiO_2_ would be formed on the surface of laser-fabricated microstructures. Then, anatase TiO_2_ would be synthesized with a hydrothermal reaction. Also, Ag nanoparticles can be decorated on the surface of TiO_2_ flower-like structures.

**Figure 8:**
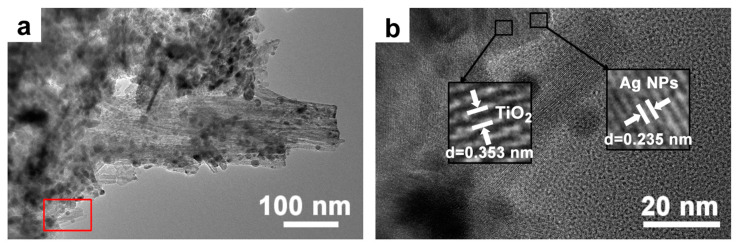
TEM and HRTEM pictures of Ag-TiO_2_-10. (**a**) Transmission electron microscope image of Ag-TiO_2_-10. (**b**) High-resolution transmission electron microscopy image of the red-boxed region in (**a**).

TEM and HRTEM analyses of Ag-TiO_2_-10 reveal that the surface of the TiO_2_ flower-like structure is closely bound to Ag nanoparticles with diameters ranging between 5 and 8 nm (in Figure 8). This observation aligns with the findings from the EDS results. The HRTEM image presented in Figure 8b highlights two distinct lattice stripe spacings: 0.357 nm and 0.285 nm. These measurements correspond to the (101) crystal plane of anatase TiO_2_ and the (102) crystal plane of Ag, respectively. This evidence further confirms that the TiO_2_ produced via the hydrothermal method is of the anatase phase.

### 3.4. Characterization of Photocatalytic Properties of Ag-TiO_2_ Micro-/Nanocomposite Structures

**Figure 9:**
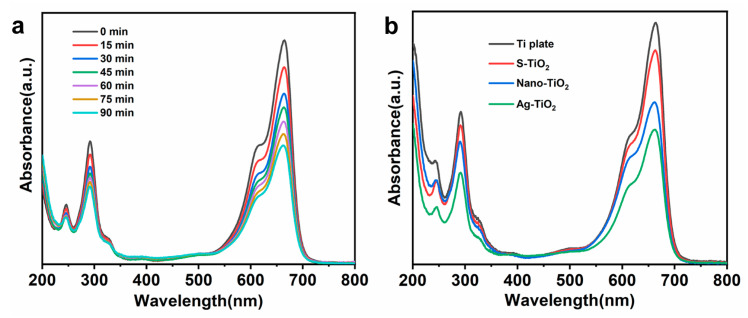
Photocatalytic ability test. (**a**) Absorption spectra of MB solution in the presence of Ag-TiO_2_ at different light times. (**b**) Absorption spectra of MB solutions in the presence of Ti plate, S-TiO_2_, nano-TiO_2_, and Ag-TiO_2_ after 90 min.

MB is commonly used to simulate typical pollutants encountered in everyday life. The photocatalytic degradation capabilities of various samples were assessed by evaluating the degradation of the MB solution under simulated sunlight. Figure 9a presents the ultraviolet–visible light absorption spectra of the MB solution when subjected to Ag-TiO_2_ over varying durations. As the irradiation time increased, the characteristic absorption peak of the MB solution at 664 nm decreased, thus suggesting that Ag-TiO_2_ can catalyze MB solution degradation under simulated sunlight. TiO_2_ with a flat surface structure exhibited negligible catalytic degradation of MB when exposed to sunlight. In this study, the femtosecond laser processing technology created abundant micro-/nanosurface structures on S-TiO_2_, amplifying the contact area between TiO_2_ and the MB solution and thereby enhancing the material’s photocatalytic degradation capability. Introducing Ag to the structure reduced the bandgap energy and expanded the catalytic effect into the visible spectrum. Simultaneously, since the atomic radius of Ag significantly surpasses that of Ti, the interface primarily comprised Ag-O-Ti. The Fermi level of Ag is below that of TiO_2_, enabling the photogenerated electrons in TiO_2_ to be injected into Ag during the photocatalytic reaction. This electron transfer diminishes the chance of electron–hole recombination in TiO_2_, bolstering its photocatalytic efficacy. Figure 9b displays the absorption spectra of various samples after 90 min in the MB solution. Since the Ti sheet has a negligible degradation effect on MB, Ti can be used as a reference. As shown in Figure 9b, the catalytic degradation effect of S-TiO_2_ is superior to that of the Ti plate. S-TiO_2_ has a catalytic effect on MB due to the formation of micro-nanostructures on the surface after femtosecond laser processing, which are accompanied with secondary micro-nanoparticles on the surface of the structural units, resulting in an increase in the specific surface area. Nano-TiO_2_ is based on S-TiO_2_ to prepare uniform TiO_2_ nanosheets to form a 3D structure, which increases the contact area with MB and therefore enhances the catalytic effect. Ag-TiO_2_ corresponds to the highest peak absorbance because the doping of Ag nanoparticles increases the band gap and the catalytic effect is no longer confined to the UV region but expands to the visible region, thus providing the best catalytic effect. Evidently, Ag-TiO_2_ demonstrated the most potent catalytic performance. The dual effects significantly augmented the photocatalytic performance of Ag-TiO_2_, registering the lowest absorption peak within the same timeframe.

**Figure 10:**
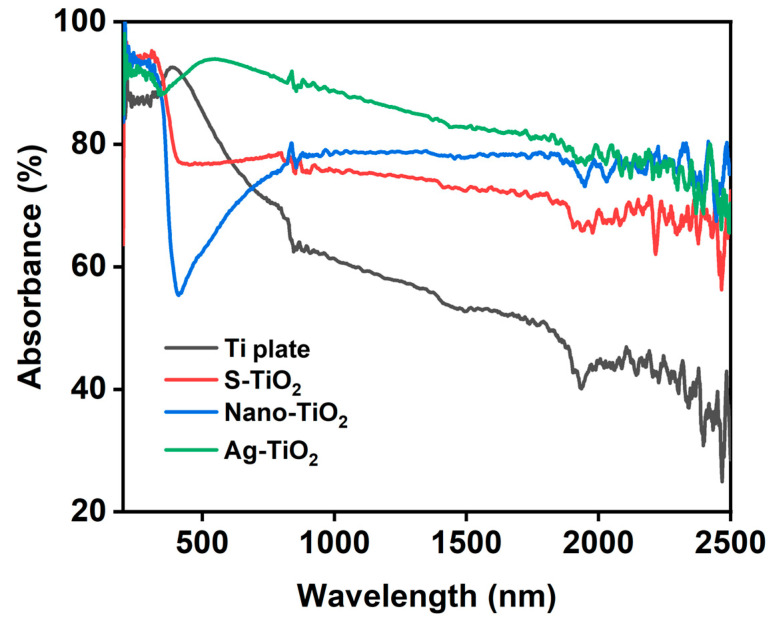
The absorbance of the different treated samples, including untreated Ti plate, S-TiO_2_, nano-TiO_2_, and Ag-TiO_2_.

The absorbance test indicates the above conclusion well, as shown in Figure 10. The untreated titanium plate has the lowest absorbance, so the photocatalytic effect is the worst. After laser processing, the titanium plate is rich in micro- and nanostructures, and due to the geometrical light trapping effect and equivalent refractive index effect, the absorbance is significantly enhanced, so the photocatalytic effect is also improved. Then, after the hydrothermal reaction, the TiO_2_ crystal phase on the surface of the sample was further enriched, but at the same time, the geometric light trapping effect in the short-wave region was affected, so that the absorbance of S-TiO_2_ had a decrease in the short-wave region and an increase in the long-wave region compared with that of nano-TiO_2_. The feedback on the photocatalytic effect is also a slight decrease in the short-wave region and an increase in the long-wave region. Finally, Ag nanoparticles were compounded, and the best photocatalytic effect was obtained due to the characteristic absorption peaks of Ag, i.e., the absorption enhancement brought about with the localized plasma resonance absorption. According to Figure 10, Ag-TiO_2_ on the laser-processed sample has a higher absorbance than other samples.

## 4. Conclusions

In this study, an Ag-TiO_2_ nanoplate composite catalyst was synthesized via a three-stage procedure, and its photocatalytic efficiency was subsequently evaluated. Utilizing femtosecond laser processing, micro-/nanogrid structures were crafted onto the Ti surface, serving as the foundation for the Ag-TiO_2_. By fine-tuning the laser power, processing speed, and duration, a textured surface topology emerged, culminating in a uniform prismatic structure boasting an extensive specific surface area.

Following this, TiO_2_ was developed on the S-Ti surface via a hydrothermal approach. Variations in the NaOH reaction concentration led to progressive morphological shifts, transitioning from nanowires to expansive nanosheets. Subsequently, Ag nanoparticles were introduced onto these nanowires via a low-temperature AgNO_3_ reduction. The density and spread of these Ag nanoparticles can be modulated by altering the processing duration.

The findings underscored that femtosecond laser processing of micro-/nanostructures enhances the specific surface area, furnishing additional reaction sites beneficial for photocatalytic reactions. The incorporation of Ag nanoparticles adeptly broadened the TiO_2_’s light absorption spectrum, stretching into the visible light domain. Under simulated visible light exposure, the Ag-TiO_2_ composite photocatalyst demonstrated a tripling in its MB degradation capacity compared with S-TiO_2_. After 90 min, this photocatalyst still exhibited commendable catalytic prowess.

## Data Availability

Not applicable.
